# Konsensuspapier zur standardisierten Labordiagnostik der chronischen Nierenerkrankung (CKD) im Kontext kardiovaskulär-renal-metabolischer Gesundheit (ÖGLMKC, ÖQUASTA, ÖGN): Konsensuspapier der Österreichischen Gesellschaft für Laboratoriumsmedizin und Klinische Chemie (ÖGLMKC), der Österreichische Gesellschaft für Qualitätssicherung und Standardisierung medizinisch-diagnostischer Untersuchungen (ÖQUASTA) und der Österreichischen Gesellschaft für Nephrologie (ÖGN)

**DOI:** 10.1007/s00508-026-02749-1

**Published:** 2026-06-12

**Authors:** Kathrin Eller, Thomas Koller, Christoph Buchta, Markus Exner, Georg Greiner, Andrea Griesmacher, Karina Hellbert, Alireza Karimi, Christoph Mache, Marcus Säemann, Georg Mustafa

**Affiliations:** 1 Österreichische Gesellschaft für Nephrologie (ÖGN), Graz, Österreich; 2 Klinische Abteilung für Nephrologie, Medizinische Universität Graz, Graz, Österreich; 3 Österreichische Gesellschaft für Laboratoriumsmedizin und Klinische Chemie (ÖGLMKC), Wien, Österreich; 4 Medilab Dr. Mustafa, Dr. Richter Labor für medizinisch-chemische und mikrobiologische Diagnostik GmbH, Salzburg, Österreich; 5 Österreichische Gesellschaft für Qualitätssicherung und Standardisierung medizinisch-diagnostischer Untersuchungen (ÖQUASTA), Wien, Österreich; 6 Labors.at, 1210 Wien, Österreich; 7 Ihr Labor, Ordinationsgemeinschaft für Labordiagnostik und Mikrobiologie, Wien, Österreich; 8 Zentralinstitut für Medizinische und Chemische Labordiagnostik (ZIMCL), Universitätskliniken Innsbruck, Innsbruck, Österreich; 9 Maybach Bechter Hellbert Rechtsanwälte, Wien, Österreich; 10 Klinische Abteilung für allgemeine Pädiatrie, Medizinische Universität Graz, Graz, Österreich; 11 Österreichische Gesellschaft für Kinder- und Jugendheilkunde (ÖGKJ), Innsbruck, Österreich; 12 6. Medizinische Abteilung mit Nephrologie & Dialyse, Klinik Ottakring, Wien, Österreich; 13 Medizinische Fakultät, SFU, Wien, Österreich

**Keywords:** Albumin-Kreatinin-Ratio (uACR), Glomeruläre Filtrationsrate, Serum-Kreatinin, Cystatin C, Kardiovaskuläres Risiko, Albumin-to-creatinine ratio (uACR), Glomerular filtration rate, Serum creatinine, Cystatin C, Cardiovascular risk

## Abstract

Dieses Konsensuspapier, erstellt von der Österreichischen Gesellschaft für Laboratoriumsmedizin und Klinische Chemie (ÖGLMKC), der Österreichische Gesellschaft für Qualitätssicherung und Standardisierung medizinisch-diagnostischer Untersuchungen (ÖQUASTA) und der Österreichischen Gesellschaft für Nephrologie (ÖGN), beschreibt standardisierte Ansätze zur Labordiagnostik der chronischen Nierenerkrankung (CKD) im Kontext der kardiovaskulär-renalen-metabolischen Gesundheit. Die CKD stellt eine bedeutende Herausforderung für das Gesundheitssystem dar, insbesondere aufgrund der geringen Erkrankungskenntnis der Betroffenen sowie der hohen Morbidität, Mortalität und Kosten.

Im Fokus stehen zwei zentrale diagnostische Parameter – die geschätzte glomeruläre Filtrationsrate (eGFR) und die Albumin-Kreatinin-Ratio im Urin (uACR) – als Grundlage für Früherkennung, Diagnosestellung und Verlaufsüberwachung. Das Papier identifiziert bestehende Defizite, insbesondere die unzureichende Nutzung der uACR im Screening sowie Probleme durch fehlende Standardisierung und methodische Heterogenität.

Es werden konkrete Empfehlungen zur analytischen und präanalytischen Standardisierung, zu einheitlichen Berechnungsmethoden insbesondere die Empfehlung zur Verwendung der EKFC-Formel zur Berechnung der eGFR, zur strukturierten Befunddarstellung sowie zur Integration von Risikobewertungen wie der Kidney Failure Risk Equation (KFRE) gegeben. Darüber hinaus werden Maßnahmen zur Qualitätssicherung, einschließlich externer Ringversuche, sowie die Bedeutung harmonisierter Befundformate für die klinische Interpretation hervorgehoben.

Abschließend fordert das Konsensuspapier die systematische Integration der CKD-Früherkennung in die Primärversorgung, eine verbesserte Abrechenbarkeit diagnostischer Leistungen und eine stärkere digitale Integration von Labordaten. Ziel ist es, die Früherkennung zu verbessern, die Vergleichbarkeit von Befunden sicherzustellen und die Versorgung von Patient:innen nachhaltig zu optimieren.

## Abstract (en)

This consensus paper, jointly developed by the Austrian Society of Laboratory Medicine and Clinical Chemistry (ÖGLMKC), the Austrian Society for Quality Assurance and Standardization of Medicinal Diagnostics (ÖQUASTA), and the Austrian Society of Nephrology (ÖGN), outlines standardized approaches to the laboratory diagnosis of chronic kidney disease (CKD) within the framework of cardiovascular-renal-metabolic health. CKD represents a major public health burden, with low awareness among affected individuals and significant implications for morbidity, mortality, and healthcare costs.

The document emphasizes the central role of two key laboratory parameters—the estimated glomerular filtration rate (eGFR) and the urinary albumin-to-creatinine ratio (uACR)—for early detection, diagnosis, and monitoring of CKD. It highlights current gaps in implementation, particularly the underuse of uACR in routine screening as well as challenges arising from methodological heterogeneity and lack of standardization.

The consensus provides detailed recommendations for analytical and preanalytical standardization, uniform calculation methods recommending the EKFC equation to evaluate eGFR, structured reporting, and the integration of risk prediction tools such as the kidney failure risk equation (KFRE). It further addresses quality assurance measures, including external proficiency testing, and stresses the importance of harmonized reporting formats to improve clinical interpretation.

Finally, the paper calls for systematic integration of CKD screening into primary care, improved reimbursement structures, and enhanced digital integration of laboratory data. The overarching goal is to improve early detection, ensure comparability of results, and strengthen patient care across healthcare settings.

## 1. Präambel und Zielsetzung

Die chronische Nierenerkrankung (chronic kidney disease, CKD) zählt zu den häufigsten nicht-übertragbaren Erkrankungen und betrifft in Österreich schätzungsweise 921.000 bis 1,04 Mio. Menschen [1]. Weniger als 10 % der Betroffenen wissen von ihrer Erkrankung [2]. Sie ist in der Regel Folge anderer Erkrankungen – ein großer Anteil ist dabei auf klassische Zivilisationskrankheiten wie Diabetes mellitus Typ 2 zurückzuführen [3]. Früherkennung und frühzeitige Behandlung können das Fortschreiten erheblich verlangsamen, Komplikationen wie kardiovaskuläre Ereignisse, Hospitalisierungen, Nierenersatztherapie und damit Morbidität und Mortalität der Betroffenen signifikant reduzieren und so die gesundheitsökonomische Belastung verringern [2, 4].

Die Früherkennung (risikobasiertes Case-Finding), Diagnosestellung und die Verlaufsüberwachung stützen sich auf zwei berechnete Schlüsselparameter – die geschätzte glomeruläre Filtrationsrate (eGFR; berechnet entweder auf Basis der Kreatinin-Konzentration [eGFRcr] oder der Kreatinin- und Cystatin-C-Konzentration [eGFRcr-cys] im Serum bzw. Plasma) und die Albumin-Kreatinin-Ratio (uACR; berechnet aus der Albumin- und Kreatinin-Konzentration im Harn). Beide Schlüsselparameter sind einfach, zuverlässig und kosteneffizient bestimmbar und bilden die Grundlage für die internationale CKD-Klassifikation. In Österreich ist die routinemäßige Bestimmung in Risikopopulationen – insbesondere der uACR – bislang jedoch nur unzureichend umgesetzt [3]. Zusätzlich erschweren Unterschiede in Messmethoden und Kalibration die Vergleichbarkeit und klinische Interpretierbarkeit diagnostischer Schlüsselparameter der CKD.

Dieses Konsensuspapier formuliert eine gemeinsame Position von ÖGLMKC, ÖQUASTA und ÖGN zur standardisierten Labordiagnostik, Befunddarstellung und Qualitätssicherung für die Früherkennung, Diagnosestellung und Verlaufsüberwachung der CKD. Ziel des Statements ist es, die analytische Qualität, Vergleichbarkeit und klinische Interpretierbarkeit von Laborbefunden zu verbessern und damit die Früherkennung, Steuerung und Versorgung von Patient:innen mit CKD in der ambulanten Versorgung – insbesondere in der Primärversorgung, wo die meisten CKD-Patient:innen betreut werden – zu stärken.

## 2. Medizinisch-wissenschaftlicher Hintergrund

Die CKD wurde gemäß der WHO-Resolution „Reducing the burden of noncommunicable diseases through promotion of kidney health and strengthening prevention and control of kidney disease“, veröffentlicht im Mai 2025, als eine der sechs nichtübertragbaren Erkrankungen mit hoher globaler Priorität eingestuft [5]. Prognosen zufolge wird die CKD bis 2040 zu den fünf häufigsten Todesursachen zählen [6]. Auch in Österreich stellt die CKD bereits ein erhebliches Gesundheitsproblem dar: 2017 war die CKD für rund 2600 bis 2900 Todesfälle direkt verantwortlich [7]. Eine aktualisierte Schätzung für 2023 beziffert diese Zahl auf 3340 bis 4440 Todesfälle [1]. Rechnet man kardiovaskuläre Todesfälle hinzu, die auf eingeschränkte Nierenfunktion zurückzuführen sind, ergibt sich für 2017 eine geschätzte Gesamtbelastung von etwa 5600 bis 6400 Todesfällen [7].

Die CKD ist eng mit Herz- und Stoffwechselerkrankungen („Cardiovascular-Kidney-Metabolic“, CKM) verknüpft [8]. Neben der gesundheitlichen Bedeutung ist auch die ökonomische Belastung beträchtlich – Analysen zeigen, dass die Kosten der CKD-Versorgung in Europa in ähnlicher Größenordnung liegen wie jene für onkologische Erkrankungen oder Diabetes mellitus [9].

In den letzten Jahren haben sich die therapeutischen Möglichkeiten deutlich erweitert. SGLT2-Inhibitoren, nichtsteroidale Mineralokortikoid-Antagonisten und GLP-1-Rezeptoragonisten verbessern nachweislich und deutlich die Prognose von CKD-Patient:innen. Diese verlangsamen die Progression der Nierenerkrankung und kardiorenale Endpunkte wie kardiovaskuläre Sterblichkeit oder Herzinsuffizienz-Hospitalisierungen werden reduziert, zudem werden relevante Endpunkte wie das Auftreten von akutem Nierenversagen oder Hospitalisierung günstig beeinflusst [2, 10, 11]. Diese Fortschritte setzen jedoch eine rechtzeitige Diagnosestellung voraus.

Zu den größten Risikopopulationen für die Entwicklung einer CKD zählen Menschen mit Diabetes mellitus, arterieller Hypertonie und kardiovaskulären Erkrankungen (Herzinsuffizienz, koronare Herzkrankheit, periphere arterielle Verschlusskrankheit, zerebrovaskuläre Erkrankungen), diese umfassen in Österreich rund ein Viertel der Bevölkerung [3]. Internationale wie nationale Leitlinien empfehlen für diese Gruppen eine regelmäßige Früherkennung mittels eGFR und uACR [12] – bei Diabetes jährlich, bei den anderen Risikopopulationen alle zwei Jahre [13]. Auch systematische Übersichtsarbeiten zur Kosteneffektivität sprechen für die Früherkennung in Risikopopulationen wie Diabetes mellitus und arterielle Hypertonie [14–17].

Internationale und nationale Daten zeigen, dass 60 bis 74 % der Betroffenen ausschließlich über die Bestimmung der uACR identifiziert werden können – nicht über die eGFR alleine [18–20]. Ergebnisse der THOMAS-Studie zeigen darüber hinaus, dass ein uACR-basiertes Screening auch bislang unerkannte Hypertonie, Diabetes und weitere kardiovaskuläre Risikofaktoren erfassen kann [21]. Die Albuminurie-Diagnostik spielt daher – als besonders sensitiver gemeinsamer Marker renaler und kardiovaskulärer Schädigung – eine Schlüsselrolle bei der systematischen, niederschwelligen Früherkennung in der Primärversorgung.

**Abb. 1:**
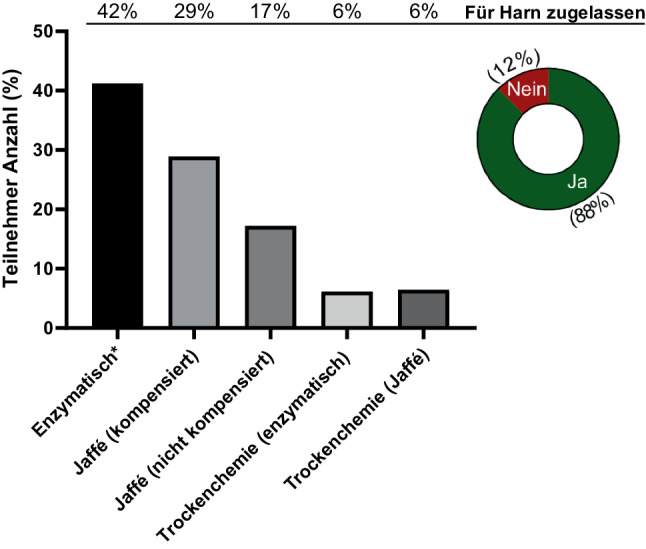
Aufschlüsselung der von Teilnehmern verwendeten Methoden für die Kreatininbestimmung (ÖQUASTA Ringversuch Nr. 265 (Jahr: 2025)). (Dargestellt ist der prozentuale Anteil der Ringversuchsteilnehmer je eingesetzter Methode. Die Prozentwerte sind *oberhalb der Balken* angegeben. Das *rechts* dargestellte *Ringdiagramm* zeigt den Anteil der Teilnehmer, die eine für die Kreatininbestimmung im Harn zugelassene Methode anbieten. Bei der angeführten Kategorie Trockenchemie (enzymatisch oder Jaffé) handelt es sich ausschließlich um POCT-Geräte. *Ein Trockenchemie Gerät, welches kein POCT-Gerät ist, wurde der Kategorie „enzymatisch“ zugeordnet. Anmerkung: Anwender, die nicht an diesem ÖQUASTA Ringversuch teilgenommen haben, werden in dieser Abbildung nicht berücksichtigt)

**Abb. 2:**
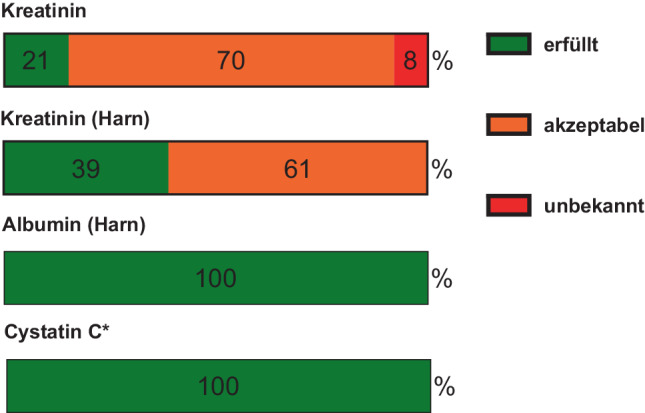
Prozentsatz der Anwender, deren Testsysteme Kalibrationsstandard Empfehlungen (Goldstandard) einhalten. (Dargestellt ist der prozentuale Anteil der Teilnehmer, die die aktuellen Empfehlungen zum Kalibrationsstandard erfüllen (*grün*) (Kreatinin: NIST SRM 967a/b für Serum, 3667 für Harn; Cystatin C: ERM-DA471, Albumin (Harn): ERM-DA/CRM 470(k)), einen akzeptablen, jedoch nicht den neuesten Standard verwenden (*orange*) oder einen unbekannten Standard einsetzen (*rot*) – jeweils für die Bestimmung von Kreatinin, Kreatinin im Harn, Cystatin C und Albumin im Harn. Cystatin C und Albumin (Harn) enthalten keine POCT-Geräte. *Cystatin C Ergebnisse stellen keine direkten Ergebnisse aus einem Ringversuch dar, sondern wurden durch Recherche der in Österreich verfügbaren Hersteller ermittelt)

Damit diese niederschwellige Früherkennung in der Versorgung zuverlässig umgesetzt und Befunde über Einrichtungen hinweg vergleichbar interpretiert werden können, ist eine hohe Standardisierung der zugrundeliegenden Messgrößen erforderlich. Ringversuche der ÖQUASTA zeigen beispielhaft, dass österreichweit sehr unterschiedliche Methoden verwendet werden (siehe Abb. 1). Nicht alle der in Österreich verwendeten Methoden zur Bestimmung der zugrundeliegenden Analyten Kreatinin, Cystatin C und Albumin entsprechen dem derzeit empfohlenen internationalen Goldstandard. Zusätzlich kommen verschiedenste, zum Teil veraltete Kalibrationsstandards zur Anwendung (siehe Abb. 2 und 3). Diese Heterogenität der Testsysteme und der verwendeten Kalibrationsstandards beeinträchtigt die leitliniengerechte Interpretation und Vergleichbarkeit der Befunde (Abb. 4).

**Abb. 3:**
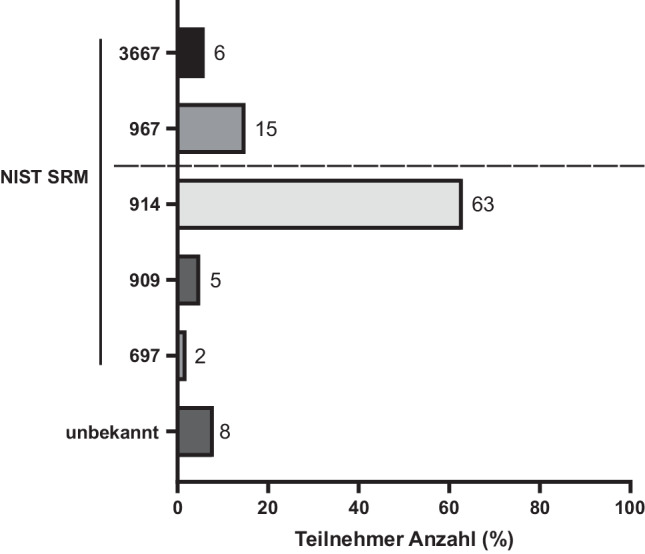
Aufschlüsselung der von Teilnehmern verwendeten Standards zur Kreatininbestimmung in Österreich (Ringversuch Nr. 265 – 2025). (Dargestellt ist der prozentuale Anteil der Teilnehmer, die den angegeben Kalibrationsstandard zur Kreatininbestimmung verwenden. *Über der horizontal strichlierten Linie* finden sich die derzeit aktuellen Goldstandard-Materialien, *darunter* weitere, ältere, jedoch akzeptable Kalibrationsstandards. Testsysteme mit unbekannten Kalibrationsstandards sollten nicht verwendet werden)

**Abb. 4:**
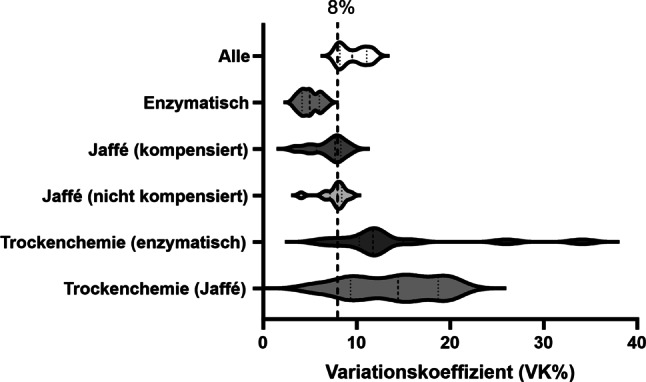
Darstellung der Variationskoeffizienten der von den Teilnehmern der Ringversuche verwendeten Methoden zur Kreatininbestimmung. (Die Abbildung zeigt die Variationskoeffizienten der ÖQUASTA-Ringversuchsteilnehmer für die einzelnen Methoden der Kreatininbestimmung. Eingeschlossen sind sechs Ringversuche aus den Jahren 2023–2025 mit jeweils zwei Proben. Die *vertikal gestrichelte Linie* bei 8 % VK markiert die aktuell empfohlene Grenze. Median und Quartile sind innerhalb der Violin-Plots durch *gestrichelte Linien* dargestellt. Bei der angeführten Kategorie Trockenchemie (enzymatisch oder Jaffé) handelt es sich ausschließlich um POCT-Geräte (laut Herstellerangaben))

## 3. Diagnostische Schlüsselparameter

### 3.1. Überblick

Wie oben beschrieben stützen sich Früherkennung, Diagnosestellung sowie die Verlaufsüberwachung der CKD auf die eGFR und die uACR. Darüber hinaus spielt bei einer eGFR < 60 ml/min/1,73 m^2^ die Berechnung des Risikos für ein terminales Nierenversagen mittels Kidney Failure Risk Equation (KFRE) (5-Jahres- sowie 2‑Jahres-Risiko) klinisch eine zunehmend entscheidende Rolle und wird von nationalen und internationalen Leitlinien empfohlen (siehe z. B. KDIGO 2024 2.2.1) [2, 13].

Für die Diagnose einer CKD müssen eGFR und/oder uACR seit mindestens drei Monaten im pathologischen Bereich liegen (i.e. zwei Messungen im Abstand von drei Monaten mit eGFR < 60 ml/min/1,73 m^2^ und/oder uACR ≥ 30 mg/g). Die Diagnose kann daher nicht anhand einer einzelnen Bestimmung gestellt werden. Liegen allerdings entsprechende Vorbefunde mit pathologischen Werten vor, kann die Diagnosestellung sofort erfolgen.

### 3.2. eGFR

- Angabe: ml/min/1,73 m^2^ mit Zuordnung zur KDIGO-GFR-Kategorie G1–G5
- Referenzbereich: ≥ 60 ml/min/1,73 m^2^

Bei Erwachsenen und Kindern (≥ 2 Jahre alt) wird die Berechnung der eGFRcr aus Serum/Plasma-Kreatinin nach EKFC 2021 [22] oder, sofern Cystatin C verfügbar ist, die Berechnung der eGFRcr-cys aus dem arithmetischen Mittel der eGFRcr nach dem *European Kidney Function Consortium (*EKFC) 2021 und der eGFRcys (aus Serum/Plasma Cystatin C) nach EKFC 2023 empfohlen [2, 22–24] – Formeln siehe Tab. 1.

**Tab. 1:** Empfohlene eGFR-Formeln

Name	Alter	Verhältnis zum Q‑Wert	Formel	Details
eGFRcr	2–40	Cr/Q < 1	107,3 × (Cr/Q)^−0,322^	Q‑Werte siehe Beschreibung unter der Tabelle
	2–40	Cr/Q ≥ 1	107,3 × (Cr/Q)^−1,132^	
	> 40	Cr/Q < 1	107,3 × (Cr/Q)^−0,322^ × 0,990^(Alter^ ^−^ ^40)^	
	> 40	Cr/Q ≥ 1	107,3 × (Cr/Q)^−1,132^ × 0,990^(Alter^ ^−^ ^40)^	
eGFRcys	18–40	Cys/0,83 < 1	107,3 × (Cys/0,83)^−0,322^	–
	18–40	Cys/0,83 ≥ 1	107,3 × (Cys/0,83)^−1,132^	
	> 40	Cys/0,83 < 1	107,3 × (Cys/0,83)^−0,322^ × 0,990^(Alter^ ^−^ ^40)^	
	> 40	Cys/0,83 ≥ 1	107,3 × (Cys/0,83)^−1,132^ × 0,990^(Alter^ ^−^ ^40)^	
	> 50	Cys/Q < 1	107,3 × (Cys/Q)^−0,322^ × 0,990^(Alter^ ^−^ ^40)^	Q = 0,83 + 0,005 × (Alter − 50)
	> 50	Cys/Q ≥ 1,0	107,3 × (Cys/Q)^−1,132^ × 0,990^(Alter^ ^−^ ^40)^	Q = 0,83 + 0,005 × (Alter − 50)
eGFRcr-cys	≥ 2	–	(eGFRcr + eGFRcys)/2	–

**Q‑Werte für eGFRcr: **Für weiße Patient:innen im Alter > 25 Jahre gilt: Männer: Q = 0,90 mg/dL, Frauen: Q = 0,70 mg/dL. Für Patient:innen im Alter von 2 bis 25 Jahre gilt: Männer: ln(Q) = 3,200 + 0,259 × Alter − 0,543 × log(Alter) − 0,00763 × Alter^2^ + 0,0000790 × Alter^3^, Frauen: ln(Q) = 3,080 + 0,177 × Alter − 0,223 × log(Alter) − 0,00596 × Alter^2^ + 0,0000686 × Alter^3^. Q muss für diese Patient:innen von μmol/L in mg/dL umgerechnet werden: Q/88,4

Quellen: EKFC 2023 (Supplement: „Section S6. Overview of eGFR-equations“) [23], EKFC 2021 [22]

Zumindest im Rahmen von Therapieentscheidungen, die von der GFR abhängen (z. B. Dosierung von Arzneimitteln), oder wenn Zweifel an der Richtigkeit des Serum/Plasma-Kreatinins bestehen, wird empfohlen zusätzlich Cystatin C zu bestimmen und die eGFRcr-cys (statt der eGFRcr) zu berechnen [2, 13] (siehe auch Tab. 2) – insbesondere bei:

- Veränderungen des körperlichen Habitus/der Muskelmasse (z. B. Essstörungen wie Anorexie, gezieltes Krafttraining oder Bodybuilding, Amputationen, Rückenmarksverletzung mit Para- oder Tetraplegie/-parese, Adipositas Grad III),
- Veränderungen der Diät (z. B. eiweißreiche Diät, Kreatinpräparate oder Vorstufen),
- spezifischen Erkrankungen (z. B. Herzinsuffizienz, Leberzirrhose, katabole konsumierende Erkrankungen, Erkrankungen mit Muskelabbau) und Medikamenten (z. B. Steroide).

**Tab. 2:** Indikationen zur zusätzlichen Messung von Cystatin C (eGFRcr-cys)

Indikationen zur zusätzlichen Messung von Cystatin C
*Habitus & veränderte Muskelmasse*
Gezieltes Krafttraining, Bodybuilding, Extremsport
Essstörung, z. B. Anorexie
Amputationen
Rückenmarksverletzung mit Para- oder Tetraplegie/-parese
Adipositas (vor allem BMI > 40)
*Ernährung & Lifestyle*
Niedrige Eiweißzufuhr
Hohe Eiweißzufuhr
Ketogene Diät
Vegetarische/Vegane Ernährung
Kreatin Nahrungsergänzungsmittel
*Krankheiten*
Malnutrition
Maligne Erkrankungen
Herzinsuffizienz
Zirrhose
Katabole Erkrankungen (z. B. Tuberkulose, AIDS, Hämatologische Erkrankungen)
Muskelabbau-Erkrankungen
*Medikamente*
Steroide (anabolisch, Hormone)
Breitbandantibiotika mit reduzierter extrarenaler Eliminierung

Adaptiert von KDIGO 2024 [2, Tab. 8]

In ausgewählten Situationen kann es sinnvoll sein, die GFR mittels Plasma- oder Urin-Clearance eines exogenen Filtrationsmarkers zu messen (siehe Tab. 3).

**Tab. 3:** Indikationen zur (direkten) Messung der GFR (mGFR)

Indikationen zur (direkten) Messung der GFR (mGFR)
*Klinische Szenarien, in denen eGFRcr-cys inakkurat oder möglicherweise falsch ist*
Katabole Zustände wie schwere Infektionen oder Entzündungen
Hoher Zell-Turnover (bei manchen Tumorerkrankungen)
Fortgeschrittene Leberzirrhose
Fortgeschrittene Herzinsuffizienz
Hochdosierte Steroidgabe
Gebrechlichkeit (Frailty)
*Klinische Szenarien, die höhere Messgenauigkeit erfordern als mit eGFRcr-cys erreichbar*
Entscheidungen über eine simultane Nierentransplantation zum Zeitpunkt einer anderen soliden Organtransplantation
Nierenspender-Evaluierung
Medikamentendosierung bei geringem therapeutischem Index oder schwere Toxizität (z. B. Chemotherapien, die renal eliminiert werden)

Adaptiert von KDIGO 2024 [2, Tab. 10]

Anmerkungen:

- KDIGO 2024 empfiehlt, innerhalb geografischer Regionen (z. B. eines Landes) dieselbe eGFR-Gleichung zu verwenden, um Vergleichbarkeit und konsistente klinische Entscheidungen zu gewährleisten [2].
- Die European Federation of Clinical Chemistry and Laboratory Medicine (EFLM) empfiehlt die Nutzung der EKFC-Formeln [25, 26]. Auch KDIGO 2024 unterstützt die Nutzung [2]. Die EKFC-Formeln wurden primär auf Basis europäischer Kohorten entwickelt und sind in Europa umfassend validiert [22, 23, 25].
- Für die EKFC-Formeln werden u. a. folgende zusätzliche Vorteile genannt: Sie liefern über den Übergang von Kindheit und Adoleszenz ins Erwachsenenalter hinweg vergleichbare eGFR-Werte. Im Vergleich zu CKD-EPI wird zudem eine Tendenz zur GFR-Überschätzung bei jungen Erwachsenen vermieden. Bei der Cystatin-C-basierten EKFC-Formel wird weder eine Ethnizitäts- noch eine Geschlechtsvariable verwendet, was insbesondere im Kontext transgeschlechtlicher Personen relevant sein kann [25].
- Sonderpopulationen mit eingeschränkter Nutzbarkeit der empfohlenen eGFR-Formeln (siehe auch Tab. 4):
  - In Kindern < 2 Jahren sind die eGFR-Formeln nicht validiert.
  - In der Schwangerschaft ist die Anwendung von eGFR-Formeln durch physiologische Hyperfiltration und Veränderungen der Körperoberfläche erschwert [2]. Die Nutzung der Kreatinin Konzentration wird empfohlen [27].
  - Für dialysepflichtige Patient:innen sind eGFR-Formeln nicht validiert.
  - Bei Zuständen ohne Steady State, wie z. B. einer akuten Nierenschädigung, sind die genannten eGFR-Formeln nicht verlässlich.
- Fehlerquellen bei der Abschätzung der eGFR umfassen laut KDIGO 2024 [2]:
  - Zustände ohne Steady State (z. B. akute Nierenschädigung (AKI)),
  - potenzielle nicht-GFR-abhängige Determinanten von Kreatinin und Cystatin C (z. B. katabole Zustände, etwa bei schweren Infektionen oder ausgeprägten entzündlichen Erkrankungen, hoher Zellumsatz wie bei bestimmten malignen Erkrankungen, fortgeschrittene Leberzirrhose oder Herzinsuffizienz, die Anwendung hochdosierter Steroide sowie ausgeprägte Gebrechlichkeit),
  - Messungenauigkeiten im höheren GFR-Bereich (aufgrund höherer biologischer Variabilität nicht-GFR-abhängiger Determinanten sowie höherer Messungenauigkeit),
  - analytische Interferenzen (siehe Tab. 5 und 6).

**Tab. 4:** Sonderpopulationen mit eingeschränkter Nutzbarkeit der empfohlenen eGFR-Formeln

Population	eGFR_CR_ nach EKFC 2021	eGFR_CR-CYS_ nach EKFC 2021 und EKFC 2023	Alternativen
*Kinder <* *2 Jahren*	Nicht validiert	Nicht validiert	*–*
*Dialyse*	Nicht validiert	Nicht validiert	
*Zustände ohne Steady-State (z.* *B. akute Nierenschädigung)*	Nicht zuverlässig	Nicht zuverlässig	*Ggfs. Nutzung validierter kinetischer eGFR-Formeln*
*Schwangerschaft*	Nicht zuverlässig	Nicht zuverlässig	*Serum/Plasma-Kreatinin-Konzentration *[27]

**Tab. 5:** Beispiele für analytische Störfaktoren der Kreatininmessung

Jaffé Methode	Enzymatische Methode
**Medikamente**	
Acetaminophen	*Lidocain*
Aspirin	*Metamizol*
Blutersatzprodukte	N‑Acetylcystein
Cephalosporine	Prolin-Stabilisatoren (in intravenösem Immunglobulin (IVIG))
Fluorescein	Phenindion
Metamizol	
Streptomycin	
**Endogene Faktoren**	
*Ascorbinsäure*	Bilirubin
*Bilirubin*	
Glukose	
Hämoglobin F	
Ketone/Ketonkörper/Ketonester	
Lipide	
**Präanalytische Faktoren**	
Bakterielle Kontamination	–
Pyruvate (z. B. von alter Blutprobe)	

*Kursiv* markierte Störfaktoren sind erfahrungsgemäß in der klinischen Routine besonders bedeutsam. Zusätzlich zu den in der Tabelle aufgeführten allgemeinen Störfaktoren finden sich spezielle, gerätespezifische Störfaktoren in den Beipacktexten der Hersteller

Adaptiert von KDIGO 2024 [2, Tab. 12]

**Tab. 6:** Beispiele für analytische Störfaktoren der Cystatin-C-Messung

CYSTATIN C
Rheumafaktor
Paraprotein [37]
Lipämie (Triglyceride)
Bilirubin (Ikterus)
Hämolyse
High-Dose-Hook-Effekt

**Tab. 7:** Beispiele für analytische Störfaktoren der Albumin-Messung

Albumin Harn
Bakterielle Kontamination
High-Dose-Hook-Effekt
Sammelgefäß-Adsorption [38]
pH-Wert < 5,0
*Je nach verwendeter Methode/Gerät*
Hämolyse [39]
Hyperbilirubinämie [40]

### 3.3. uACR

- Angabe: mg/g mit Zuordnung zur KDIGO-Albuminurie-Kategorie A1–A3
- Referenzbereich: < 30 mg/g

Zur Berechnung der uACR wird die Harn-Albumin-Konzentration durch die Harn-Kreatinin-Konzentration dividiert. Qualitätsgesicherte quantitative Messungen von Albumin und Kreatinin im Harn in einem medizinisch-diagnostischen Labor werden empfohlen.

Anmerkungen:

- Produkte für patientennahe Tests^1^ (Point-of-Care-Testing, POCT) können laut KDIGO 2024 insbesondere bei eingeschränktem Zugang zu einer zentralen Laboranalytik erwogen werden [2]. In Österreich ist eine zentrale Laboranalytik in der Regel jedoch flächendeckend und zeitlich gut verfügbar. Falls doch ein Einsatz von einem CE-zertifizierten POCT-Produkt notwendig werden sollte, liegt es in der Verantwortung der durchführenden Ärzt:in, die Einhaltung der unten angeführten Leitlinienvorgaben sicherzustellen, ggf. in Abstimmung mit den von ihm/ihr in Anspruch genommenen medizinisch-diagnostischen Laboratorien.
- Bei Einsatz von diesen POCT-Produkten sind präanalytische, analytische und postanalytische Qualitätskriterien inkl. externer Qualitätskontrolle einzuhalten (siehe KDIGO 1.4.1). Zusätzlich ist die Fähigkeit, Kreatinin zu messen und eine uACR auszugeben, wichtig. Im Rahmen der Evaluierung und Entscheidung über den Einsatz eines POCT-Produktes soll (durch die niedergelassene Ärzt:in) geprüft werden, ob es bei mindestens 85 % der Personen mit signifikanter Albuminurie (uACR ≥ 30 mg/g) ein positives Ergebnis liefert (siehe KDIGO 1.4.3). Dazu ist eine Verifizierung mittels Analyse einer Kontrollprobe, die eine uACR ≥ 30 mg/g ergibt, erforderlich. [2]
- Streifentests (insbesondere semiquantitative) weisen eine deutlich geringere Sensitivität und Spezifität auf [2, 28, 29]. Laut NICE sollten Streifentests nur dann eingesetzt werden, wenn sie Albumin auch in niedrigen Konzentrationen spezifisch messen, das Ergebnis als uACR ausgeben und verifiziert wurde, dass sie eine Kontrollprobe, die eine uACR ≥ 30 mg/g ergibt, verlässlich detektieren [30]. Bei der Nutzung von Streifentests empfiehlt KDIGO 2024 die automatisierte Auslesung (siehe KDIGO 1.3.1 ii) [2]. Auffällige Streifentest-Befunde sollen durch quantitative uACR-Bestimmung (siehe KDIGO 1.3.1.2) [2] in einem medizinisch-diagnostischen Labor bestätigt werden.
- Mehrere Faktoren können die Konzentrationen von Albumin im Urin (z. B. Hämaturie (etwa postoperativ urologisch), Menstruation, körperliche Belastung, Infektionen) beeinflussen. Beispiele für analytische Störfaktoren der Albumin-Messung sind in Tab. 7 aufgelistet. Die Kreatinin-Konzentration selbst variiert interindividuell, etwa in Abhängigkeit von Geschlecht, Körpergewicht oder Proteinaufnahme (s. KDIGO 2024 Leitlinie [2, Tab. 16]).
- Albumin vs. Gesamtprotein im Harn: Insbesondere in CKD-Risikopopulationen gilt die uACR aufgrund ihrer höheren Sensitivität der uPCR (Urin-Protein-Kreatinin-Ratio) gegenüber als überlegener Test. Die Evidenzlage zu CKD und Therapieeffekten – etwa aus Diabetes- und kardiovaskulären Studien – basiert überwiegend auf der uACR, während in der Diagnostik glomerulärer Erkrankungen traditionell die uPCR verwendet wird. Dadurch ist die uACR die Standardanwendung für das CKD-Management in der ambulanten Versorgung. Die uACR erfasst jedoch nur Albumin. Bei Verdacht auf Nicht-Albumin-Proteine (z. B. Diagnostik von tubulären Störungen oder Leichtkettenerkrankungen) ist die Bestimmung der uPCR sinnvoll [13].

### 3.4. KFRE

- Angabe: in %; Anwendung nur bei eGFR < 60 ml/min/1,73 m^2^
- Referenzbereich: KFRE 5‑Jahres-Risiko < 5 %, KFRE 2‑Jahres-Risiko < 10 %

Berechnung des 2‑ und 5‑Jahres-Risikos für ein terminales Nierenversagen aus eGFR, uACR, Alter und Geschlecht (4-Variablen-Gleichung) unter Verwendung des Kalibrierungsfaktors für außerhalb Nordamerikas [2, 25, 31] – Formeln siehe Tab. 8.

**Tab. 8:** KFRE-Formeln

Name	Geschlecht	Formel
5‑Jahres-Risiko nach KFRE	Frauen	1 − 0,9365 ^ exp (−0,2201 × (Alter/10 − 7,036) + 0,2467 × (−0,5642) − 0,5567 × (eGFR/5 − 7,222) + 0,4510 × (lnACR − 5,137))
	Männer	1 − 0,9365 ^ exp (−0,2201 × (Alter/10 − 7,036) + 0,2467 × (1 − 0,5642) − 0,5567 × (eGFR/5 − 7,222) + 0,4510 × (lnACR − 5,137))
2‑Jahres-Risiko nach KFRE	Frauen	1 − 0,9832 ^ exp (−0,2201 × (Alter/10 − 7,036) + 0,2467 × (−0,5642) − 0,5567 × (eGFR/5 − 7,222) + 0,4510 × (lnACR − 5,137))
	Männer	1 − 0,9832 ^ exp (−0,2201 × (Alter/10 − 7,036) + 0,2467 × (1 − 0,5642) − 0,5567 × (eGFR/5 − 7,222) + 0,4510 × (lnACR − 5,137))

Anwendung nur bei eGFR < 60 ml/min/1,73 m^2^

Alter (in Jahren); lnACR = natürlicher Logarithmus der uACR in mg/g (siehe [41, 42])

Version „Regional Calibrated Original – non-North America“ der 4‑Variablen-Gleichung (siehe eAppendix 2 [31])

Anmerkungen:

- Die Berechnung ermöglicht eine individuelle Risikostratifizierung und dient als Entscheidungshilfe für das weitere Handlungsprozedere – einschließlich der Empfehlung zur Begutachtung in der nephrologischen Fachversorgung bzw. zur Vorstellung zur Planung einer Nierenersatztherapie.
- Da eGFR und uACR unmittelbar in die Formel eingehen, übertragen sich die oben beschriebenen Einflussfaktoren und potenziellen Fehlerquellen dieser Parameter auch auf die Berechnung der KFRE [32].
- Bei Patient:innen ≥ 80 Jahren mit fortgeschrittener CKD wurde eine Überschätzung des Risikos eines Nierenversagens durch die KFRE beschrieben [33].

## 4. Analytische und präanalytische Standardisierung

Die korrekte Interpretation der Befunde setzt eine hohe analytische und präanalytische Qualität voraus. Der Anwender hat jedenfalls die Pflicht, mit den angewendeten Methoden, inklusive deren Leistungsmerkmalen und Limitationen sowie verwendeten Standards, vertraut zu sein:

Präanalytik:

- **Serum/Plasma**: innerhalb von 12 h nach der Blutabnahme durch Zentrifugation von den Erythrozyten trennen [25].
- **Harn**: Spontanharn, idealerweise eine Mittelstrahlurinprobe aus dem ersten Morgenurin (i.e. erste Blasenentleerung nach dem Aufstehen am Morgen) oder falls nicht machbar stets dieselbe Uhrzeit in den Folgekontrollen [2, 13, 25]. Sammelharn ist eine reine Spezialanwendung und spielt in der Früherkennung der CKD keine Rolle. Proben sollten bevorzugt frisch analysiert oder können bei 4 °C bis zu 7 Tage gelagert werden [25]. Die Möglichkeit der Lagerung bei −20 °C ist abhängig vom jeweiligen Hersteller (siehe Herstellerangaben im Beipacktext).

Analytik:

- **Kreatinin**: Bevorzugung enzymatischer Methoden gegenüber Jaffé-Methoden in Harn und Serum/Plasma [2, 25]. Bei Anwendung der Jaffé Methode wird ausdrücklich empfohlen, eine kompensierte Form der Methode (d. h. Jaffé-Methode mit mathematischer Korrektur für Nicht-Kreatinin-Chromogene/Interferenzen) zu verwenden. Im Kindesalter sollten einheitlich enzymatische Methoden zur Anwendung kommen [2]. Rückführbarkeit der Kreatininmessung auf NIST-SRM 967a (ID/MS) oder gleichwertigen/höheren ID/MS Standard ist zu verwenden [25]. Anzustrebende analytische Performance: CV < 8 % (Interassayvariabilität) [34]. Kreatinin aus dem Harn: CV ≤ 12 % [35].
- **Cystatin C:** Rückführbarkeit auf ERM-DA471/IFCC empfohlen [25]. Anzustrebende analytische Performance: CV ≤ 13 %; Bias ≤ 5 % [35]. Bei Umstellung von Pre-IFCC zu IFCC Standard kommt es zu einem Methodenbedingten 17 % Anstieg der Cystatin C Werte [36].
- **Albumin:** Nur quantitative, Immunoassay-basierte Methoden (Immunoturbidimetrie, Immunonephelometrie) sollten verwendet werden (siehe oben) [25]. Kolorimetrische Farbstoffbindungsmethoden wie Bromkresolgrün (BCG) und Bromkresolpurpur (BCP) sind ungeeignet, da ihnen die erforderliche Sensitivität und Spezifität zur Detektion niedriger Albuminkonzentrationen im Harn fehlt [25]. Anzustrebende analytische und Qualitätsparameter: Albumin aus dem Harn: CV ≤ 15 % [35].

## 5. Befunddarstellung und klinische Kommunikation

Eine einheitliche, klinisch interpretierbare Befundgestaltung ist entscheidend für die systematische Früherkennung der chronischen Nierenerkrankung und die Unterstützung des Handlungsprozederes in der ambulanten Versorgung. Alle laborseitigen Umstellungen mit Auswirkung auf den Befund (Berechnung, Analytik etc.) sind dem Befundempfänger eindeutig und auf jedem einzelnen Befund für einen vom Labor definierten Zeitraum mitzuteilen. Basierend auf den in Kapitel 3 definierten Schlüsselparametern empfehlen ÖGLMKC, ÖGN und ÖQUASTA folgende standardisierte Darstellung.

### Struktur des Befundes

Empfohlen wird die gebündelte Darstellung der drei Schlüsselparameter in einem eigenen Befundabschnitt („Nierenfunktion“) mit folgenden Elementen:

- eGFR [ml/min/1,73 m^2^; gerundet auf ganze Zahl [25]] + KDIGO-GFR-Kategorie G1–G5
- uACR [mg/g; gerundet auf ganze Zahl [25]] + KDIGO-Albuminurie-Kategorie A1–A3
- KFRE-Risiko (nur bei eGFR < 60 ml/min/1,73 m^2^ ausgeben)
  - 2‑Jahres-Risiko [%]
  - 5‑Jahres-Risiko [%]

### KDIGO-Kategorien

Schwellenwerte für Ausgabe der GFR-Kategorie:

- G1: ≥ 90, G2: 60–89, G3a: 45–59, G3b: 30–44; G4: 15–29, G5: < 15 ml/min/1,73 m^2^

Schwellenwerte für Ausgabe der Albuminurie-Kategorie:

- A1: < 30, A2: 30–299, A3: ≥ 300 mg/g

### Empfohlene Standardkommentare bei Erwachsenen

Tab. 9**.**

**Tab. 9:** Es werden folgende Standardkommentare bei Erwachsenen in den spezifischen Befundkonstellationen empfohlen.

Befundkonstellation	Kommentar
G4–G5, A3 oder G3bA2	„Sofern noch nicht erfolgt, wird eine Vorstellung in der nephrologischen Fachversorgung empfohlen“
KFRE 5‑Jahres-Risiko ≥ 5 %	„Sofern noch nicht erfolgt, wird eine Vorstellung in der nephrologischen Fachversorgung empfohlen“
KFRE 2‑Jahres-Risiko ≥ 10 %	„Sofern noch nicht erfolgt, wird eine Vorstellung in der spitalsambulanten nephrologischen Fachversorgung empfohlen“
KFRE 2‑Jahres-Risiko ≥ 40 %	„Bei chronischer Nierenerkrankung ohne Hinweise auf ein akutes reversibles Geschehen wird – sofern noch nicht erfolgt – eine sofortige Vorstellung in der spitalsambulanten nephrologischen Fachversorgung zur weiteren Abklärung und Planung der therapeutischen Schritte empfohlen.“

### Weitere Empfehlungen zur Darstellung der Einzelparameter

Analyten im Blut (zur Berechnung der eGFR):

- Serum/Plasma-Kreatinin: auf zwei Dezimalstellen (mg/dL) runden [25]
- Cystatin C: auf zwei Dezimalstellen runden (mg/L) [25]
- Referenzbereiche: siehe Herstellerangaben.

Analyten im Harn (zur Berechnung der uACR):

- Albumin und Kreatinin, wenn möglich, nicht separat darstellen.
- Referenzbereiche: siehe Herstellerangaben.

## 6. Überwachung der Harmonisierung von Analytik, Ergebnisinterpretation und Befundkommentaren

Externe Qualitätsbewertungen („Ringversuche“) sind anzuraten, um neben der analytischen Performance auch die Eignung der in den einzelnen Laboren verwendeten Algorithmen für die Erstellung errechneter Parameter sowie die Harmonisierung der Verwendung von Befundkommentaren zu überprüfen.

ÖQUASTA bietet dazu das Ringversuchsprogramm „Nierenfunktionsdiagnostik“ an. Dieses inkludiert Ringversuche für die eGFRcr, eGFRcr-cys, uACR sowie 5‑Jahres- und 2‑Jahres-Risiko nach KFRE.

## 7. Implementierung und Versorgungsaspekte

Eine standardisierte CKD-Diagnostik ist nur wirksam, wenn sie **flächendeckend verfügbar und abrechenbar** ist.

Aktuell bestehen in einzelnen Bundesländern (z. B. Steiermark) Lücken in der Abrechenbarkeit der uACR-Bestimmung. Cystatin C ist überhaupt nur vereinzelt abrechenbar.

Damit die Empfehlungen wirksam umgesetzt werden können, braucht es ein gemeinsames Verständnis und abgestimmtes Handeln zwischen den medizinisch-diagnostischen Laboratorien, Sozialversicherungen und den datenführenden Systemen (z. B. ELGA).

ÖGLMKC, ÖGN und ÖQUASTA sprechen sich aus für:

Systematische, flächendeckende Früherkennung:

- Systematische Integration der CKD-Früherkennung in bestehende präventive und strukturierte Versorgungsprozesse (z. B. Versorgungsprogramme wie Therapie Aktiv, Vorsorgeuntersuchung), insbesondere bei Personen mit Diabetes, Hypertonie und/oder kardiovaskulären Erkrankungen.

Analytische Qualität:

- Qualitätsgesicherte quantitative Messungen von Albumin und Kreatinin im Harn in einem medizinisch-diagnostischen Labor.

Digitale Integration und Befundstandardisierung:

- Standardisierte Darstellung von eGFR, uACR und KFRE in ELGA, einschließlich einheitlicher Befundstruktur und Hinweisen zur Interpretation.
- Einbindung zusätzlicher digitaler Entscheidungsunterstützung zur Verbesserung von Früherkennung, Diagnostik und Versorgung der Nierenerkrankung.
- Sicherstellung der Verfügbarkeit der diagnostischen Schlüsselparameter sowie zugrundeliegenden Analyten in ELGA-Befunden – ein situatives Opt-Out für diese Parameter erscheint nicht angemessen.
- Überwachung der Harmonisierung der Nierenfunktionsdiagnostik durch nationale Ringversuche mit edukativem Fokus.

Verfügbarkeit und Abrechenbarkeit in Kassenlaboren:

- Bundesweite Abrechenbarkeit von Albumin und Kreatinin quantitativ im Harn zur Berechnung der uACR mittels der oben beschriebenen Methoden.
- Bundesweite Abrechenbarkeit von Cystatin C zumindest für definierte Indikationen (s. oben).

Vermeidung von Fehlanreizen, die zur Über- oder Unterdiagnostik führen:

- Vermeidung von Abrechnungslogiken, die zu unnötigen Laboranforderungen führen (z. B. Albumin quantitativ erst nach negativem Gesamtprotein im Harn abrechenbar – Gesamtprotein ist Spezialanwendung!).
- Vermeidung von Abrechnungslogiken, die zu Fehlanreizen bei der Früherkennungsdiagnostik der CKD führen – z. B. Degressionen, Obergrenzen

## 8. Konsensus-Empfehlungen in Kürze

**Früherkennung, Diagnosestellung und die Verlaufsüberwachung der CKD:**

- Systematische Diagnostik in der ambulanten Versorgung mittels eGFR und uACR – insbesondere in Risikopopulationen wie Diabetes mellitus, arterielle Hypertonie und kardiovaskulären Erkrankungen (Herzinsuffizienz, koronare Herzkrankheit, periphere arterielle Verschlusskrankheit, zerebrovaskuläre Erkrankungen).
- Bei eGFR < 60 ml/min/1,73 m^2^ zusätzlich systematische Ausgabe des 2‑Jahres- und 5‑Jahres-Risikos für ein terminales Nierenversagen nach KFRE.
- Im Rahmen von Therapieentscheidungen, die von der GFR abhängen (z. B. Dosierung von Arzneimitteln), oder wenn Zweifel an der Richtigkeit des Serum‑/Plasma-Kreatinins bestehen (s. Tab. 2), wird empfohlen zusätzlich Cystatin C zu bestimmen und die eGFRcr-cys (statt der eGFRcr) zu berechnen.
- Zur Bestimmung der uACR bzw. der dafür notwendigen Analyten werden qualitätsgesicherte quantitative Verfahren in einem medizinisch-diagnostischen Labor empfohlen.
- Befunde sollen einheitlich berechnet (eGFRcr nach EKFC 2021; eGFRcr-cys bei Erwachsenen aus dem arithmetischen Mittel von eGFRcr (EKFC 2021) und eGFRcr-cys (EKFC 2023); KFRE 2‑ und 5‑Jahres-Risiko mit den 4‑Variablen-Gleichungen und dem Kalibrierungsfaktor für außerhalb Nordamerikas) und dargestellt werden (empfohlene Einheiten, Referenzbereiche, Standardkommentare, KDIGO-GFR-Kategorie und KDIGO-Albuminurie-Kategorie; s. oben).
- Die CKD-Früherkennung soll in bestehende präventive und strukturierte Versorgungsprozesse (z. B. Therapie Aktiv, Vorsorgeuntersuchung) integriert und in ELGA einheitlich dargestellt werden.

**Überwachung der Umsetzung:**

- Überwachung der Umsetzung dieser Empfehlungen durch nationale Ringversuchsprogramme der ÖQASTA wird empfohlen.

**Abrechenbarkeit:**

- Abrechenbarkeit (uACR, Cystatin C) muss flächendeckend sichergestellt werden.

## Data Availability

Die Daten sind auf Anfrage verfügbar.

## Footnotes

1 Nach Artikel 2.6 der In-Vitro-Diagnostika-Verordnung versteht man darunter ein Produkt, „*das nicht für die Eigenanwendung, wohl aber für die Anwendung außerhalb einer Laborumgebung, in der Regel in der Nähe des Patienten oder beim Patienten, durch einen Angehörigen der Gesundheitsberufe bestimmt *ist“.
